# Spurious correlations in surface-based functional brain imaging

**DOI:** 10.1162/imag_a_00478

**Published:** 2025-02-18

**Authors:** Jayson Jeganathan, Nikitas C. Koussis, Bryan Paton, Richa Phogat, James Pang, Sina Mansour L, Andrew Zalesky, Michael Breakspear

**Affiliations:** School of Psychological Sciences, College of Engineering, Science and the Environment, University of Newcastle, Newcastle, NSW, Australia; Hunter Medical Research Institute, Newcastle, NSW, Australia; Mark Hughes Foundation Centre for Brain Cancer Research, University of Newcastle, Newcastle, NSW, Australia; School of Medicine and Public Health, College of Medicine, Health and Wellbeing, University of Newcastle, Newcastle, NSW, Australia; School of Psychological Sciences, Turner Institute for Brain and Mental Health, and Monash Biomedical Imaging, Monash University, Victoria, Australia; Centre for Sleep & Cognition & Centre for Translational Magnetic Resonance Research, Yong Loo Lin School of Medicine, National University of Singapore, Singapore, Singapore; Systems Lab, Department of Psychiatry, The University of Melbourne and Melbourne Health, Victoria, Australia

**Keywords:** surface, fMRI, bias, parcellation

## Abstract

The study of functional MRI (fMRI) data is increasingly performed after mapping from volumetric voxels to surface vertices. Processing pipelines commonly used to achieve this mapping produce meshes with uneven vertex spacing, with closer neighbours in sulci compared to gyri. Consequently, correlations between the fMRI time series of neighbouring sulcal vertices are stronger than expected. However, the causes, extent, and impacts of this “gyral bias” are not completely understood or widely appreciated. We explain the origins of this bias, and using*in-silico*models of fMRI data, illustrate how it leads to spurious results and leakage of anatomical cortical folding information into fMRI time series. We show that many common analyses can be affected by this bias, including test-retest reliability, fingerprinting, functional parcellations, and regional homogeneity. The recently developed onavg template partly reduces the bias but has relatively high residual variability in vertex spacing when projected to participant-specific surfaces. Finally, we outline recommendations to avoid or remedy the gyral bias.

## 1 Introduction

Surface-based analysis of functional magnetic resonance imaging (fMRI) data has several advantages over volumetric analysis, including better correspondence with cortical topology and the avoidance of artefacts from smoothing across gyral banks (Anticevic et al., 2008). In surface-based analysis, the brain surface is tessellated into a triangular mesh, represented by vertices and faces. Volumetric data are mapped onto the surface through a diffeomorphic projection from grey matter voxels to vertices (Dale et al., 1999;Fischl, 2012;Fischl, Sereno, & Dale, 1999;Fischl, Sereno, Tootell, et al., 1999).

Recent work has found that commonly used template surfaces have uneven inter-vertex distances, with large variations across different brain regions (Ciantar et al., 2022;Feilong et al., 2023). This problem is not widely appreciated and potentially impacts most surface-based analyses of fMRI data. In the present work, we demonstrate the adverse consequences of this uneven surface projection. First, we show that vertex density is higher in sulci, leading to problems associated with the relative undersampling of non-sulcal regions including gyri. Additionally, because vertex density tracks individuals’ unique cortical folding, structural information can become artefactually incorporated into fMRI, leading to spurious results in commonly used analyses.

It has been previously noted that volume-to-surface mapping of fMRI data results in correlations between neighbouring vertices that are stronger in sulci than in gyri (Ciantar et al., 2022). When random (spatially uncorrelated) noise time series in volume space are mapped onto the surface, adjacent vertices in the sulci have higher correlation than adjacent vertices in the gyri. The bias occurs due to up-sampling from volume data to high-resolution surface meshes. Here, we expand on the precise connection between uneven inter-vertex distances (Feilong et al., 2023) and inflated local correlations (Ciantar et al., 2022). We show that non-uniformly inflated local correlations arise not only from volume-to-surface mapping, but also from current implementations of subsequent surface smoothing.

The gyral bias in diffusion MRI is comparatively well understood. Tractography algorithms tend to terminate streamlines in gyri rather than sulci (Schilling et al., 2017). In contrast, the consequences of inflated nearest-neighbour correlations in sulci remain poorly understood. We demonstrate that overlooking this problem can lead to spurious results in functional parcellations and fMRI fingerprinting. The recently developed onavg template could potentially address this problem. This template was developed by iteratively adjusting vertex locations to minimise variability in inter-vertex distances across the cortex. On the standard group-average surface, this template reduces variance in inter-vertex distances by a factor of 10 (Feilong et al., 2023). However, projecting the template’s vertices to participant-specific surfaces could reintroduce greater variability. We, therefore, test whether improvement in inter-vertex distances and gyral bias is sustained in participant-specific onavg surfaces.

## 2 Methods

We used minimally preprocessed 3T and 7T resting-state fMRI data from the Human Connectome Project (HCP), aligned with MSMSulc and cleaned with independent component analysis (Andersson et al., 2003;Andersson & Sotiropoulos, 2015,^2016^;Feinberg et al., 2010;Fischl, 2012;Glasser et al., 2013;Glasser & Van Essen, 2011;Jenkinson et al., 2002,^2012^;Milchenko & Marcus, 2013;Moeller et al., 2010;Robinson et al., 2014,^2018^;Setsompop et al., 2012;Sotiropoulos et al., 2013;Van Essen et al., 2012;Woolrich et al., 2001). We used 7T movie viewing data only for supplementary analyses. Openly available de-identified data from the project were used in accordance with the HCP Data Use Agreement. This study was approved by the University of Newcastle Human Research Ethics Committee (H-2020-0443).

The first 20 participants with both 3T and 7T fMRI data were used (Supplementary Methods 1). The first participant’s data were used for single-subject visualisations, but analyses were repeated with the remaining participants. We used one resting-state scan (15 minutes) from each participant. Bi-hemispheric surface data were represented on the fsLR 32k mid-thickness triangular surface mesh. For parcellation-based analyses, the surface was subdivided into 300 parcels (Schaefer2018_300Parcels_Kong2022_17Networks) (Kong et al., 2021;Schaefer et al., 2018). Sulcal depth values were obtained from the FreeSurfer “sulc” output, which measures the height of each vertex above the average surface (Fischl, Sereno, & Dale, 1999).

Vertex pairs directly joined by a mesh edge were termed “neighbours”. Most vertices had five neighbours. For each vertex, we calculated the inter-vertex distance as the mean geodesic distance to its neighbouring vertices. We tested the relationship between vertex area and neighbour correlations. To calculate the area of each vertex’s neighbourhood, we allotted 1/3 of a mesh triangle’s area to each of its 3 vertices. A vertex’s area was calculated as the sum of allotments from all triangles to which it contributed (Fischl, 2012).

Independent random noise time series was used as surrogate data. Standard Gaussian noise time series with 500 time points were generated independently for each vertex on the fsLR 32k surface. For each vertex, the Pearson correlation between its time series and that of each neighbouring vertex was calculated. These correlations were Fisher z-transformed with the inverse hyperbolic tangent function, averaged across neighbours, and finally re-transformed back to a Pearson’s correlation. This result was termed the “local correlation” of the vertex. This is conceptually similar to regional homogeneity (ReHo), a measure of the rank correlation between the time series of neighbouring vertices (Jiang & Zuo, 2016). Inter-vertex distances and local correlations were averaged across all neighbours of a vertex to focus on across-vertex variability rather than variability within a vertex’s neighbours, following previous work (Ciantar et al., 2022). For analyses where variation at finer spatial scales was important, spatially varying variables such as local correlation were normalised by subtracting the mean value among all vertices within a 15 mm geodesic radius.

The main results used empirical or random data without additional smoothing, represented on the fsLR 32k surface. For secondary analyses requiring surface smoothing, we used a 2D Gaussian kernel (FWHM 2 mm or 4 mm) calculated with geodesic distances between vertices. The kernel was truncated beyond a certain spatial radius, because the kernel is effectively zero for large distances. The fraction of kernel integral discarded by truncation was set to 0.01. This was implemented in the*Connectome Spatial Smoothing*package (Mansour L et al., 2022). Volumetric data were projected to the surface using trilinear interpolation, nearest neighbour, or the ribbon method, implemented in nilearn version 0.10.1 (Abraham et al., 2014). Weighted Pearson’s correlation was used for all associations between two brain maps, where each vertex’s contribution was weighted by its surface area. Significance was tested using the “spin test”, a spatial permutation test with 1000 permutations implemented in*neuromaps*(Alexander-Bloch et al., 2018;Markello et al., 2022). Results were compared to the recently developed onavg template. Vertices in the standard onavg-ico32 mesh were mapped to participant-specific surfaces with Connectome Workbench (Marcus et al., 2011).

## 3 Results

### 3.1 Gyral bias in neighbourhood correlations

**Fig. 1.**
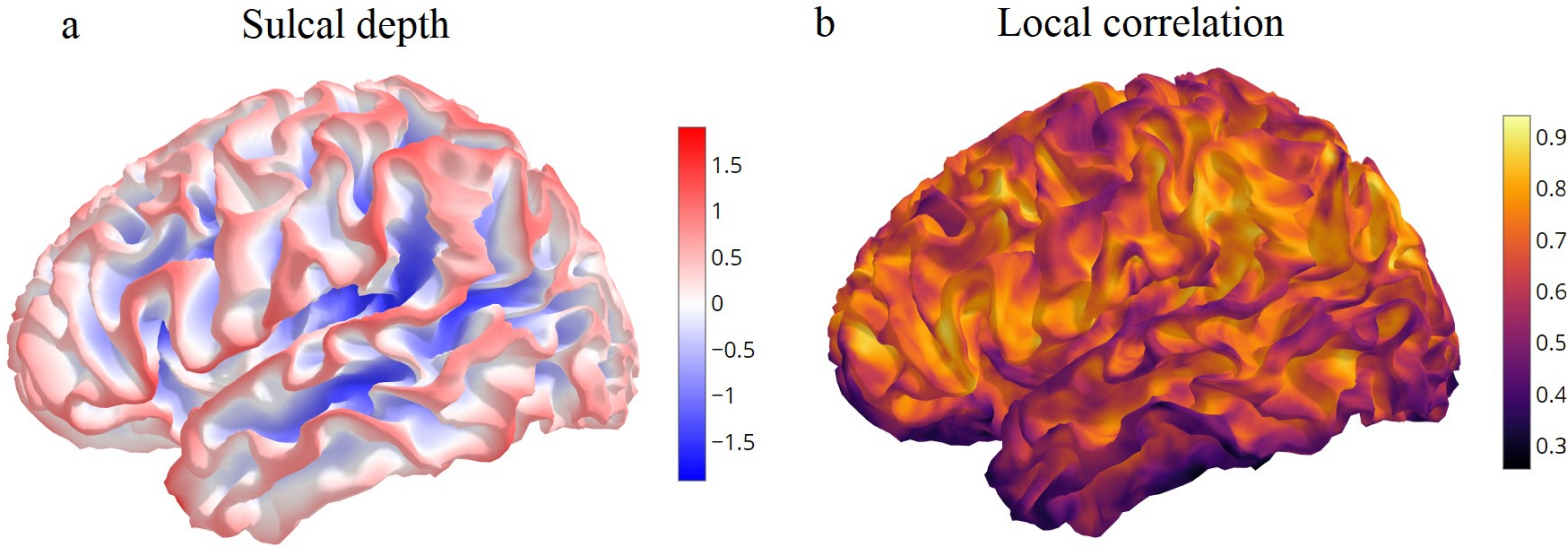
Co-variation of local fMRI correlation with sulci and gyri. Data from a single exemplar participant is shown. Spatial maps are visualised on the participant’s white matter fsLR 32k surface. (a) Sulcal depth map with gyri (red) and sulci (blue). (b) Resting-state fMRI correlation with neighbouring vertices. Correlations were not smoothed or adjusted for uneven nearest-neighbour distances.

Local correlation, which is the mean time series correlation between a vertex and its mesh neighbours, was highly biased in empirical resting-state fMRI data represented on the fsLR 32k surface. Sulcal vertices had stronger local correlation than gyral vertices (Fig. 1b). The association between local correlation and sulcal depth was significant (r = -0.340, p < 0.001).

**Fig. 2.**
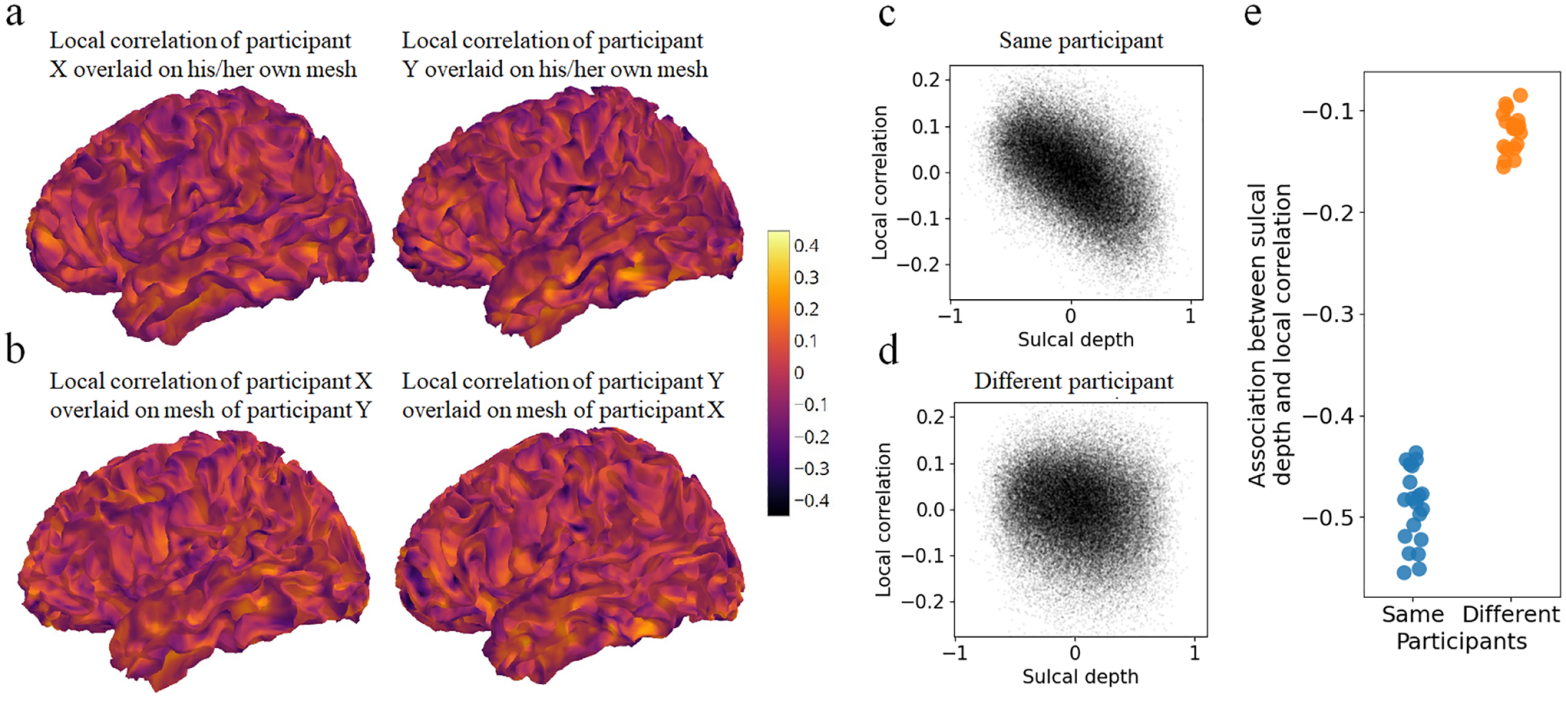
fMRI local correlations track individuals’ unique cortical folding. (a) Normalised fMRI local correlation maps for two participants overlaid on their own cortical surfaces. (b) Normalised fMRI local correlation maps overlaid on a different participant’s mesh. (c) Vertex-wise normalised local correlation values plotted against the same participant’s sulcal depth. Greater sulcal depth values indicate gyri. r = -0.508, p < 0.001. (d) Normalised local correlation plotted against a different participant’s sulcal depth. r = -0.116, p < 0.001. (e) Pearson correlation between sulcal depth map and normalised local correlation map, when the maps belonged to the same participants (blue) or different participants (orange). Each orange dot represents the correlation between local correlation of one participant and the sulcal depth map of the next participant in the sequence.

Each vertex’s local correlation value (Fig. 1b) was then normalised by subtracting the mean local correlation among all vertices within a 15 mm geodesic radius. This step isolated variation in local correlation values at the fine spatial scale. Normalised local correlation tracked each individual’s gyral crests, following the cortical folding pattern (Fig. 2a). Normalised local correlation was associated with sulcal depth (r = -0.508, p < 0.001) (Fig. 2c). Visualizing the individual’s local correlation map on the next participant’s mesh revealed an attenuated association (Fig. 2b, d) (r = -0.116, p < 0.001). The analysis was repeated with 20 participants. Participant ordering from the HCP dataset was used unchanged (subject ID 100610, 102311,…). Every participant’s sulcal depth map was more strongly associated with their own normalised local correlation map, than with the next individual’s normalised local correlation map (paired t-test, t(19) = -39.759, p < 0.001) (Fig. 2e). Therefore, fMRI neighbourhood correlations contain specific information about an individual’s unique cortical folding. The strong association between local correlations and sulcal depth was replicable with more resting-state data (1 hour instead of 15 minutes), MSMAll instead of MSMSulc, 7T movie viewing data instead of resting state, and when using the correlation between each vertex and its single nearest neighbour (Supplementary Fig. 1).

### 3.2 Uneven vertex spacing in surface meshes

**Fig. 3.**
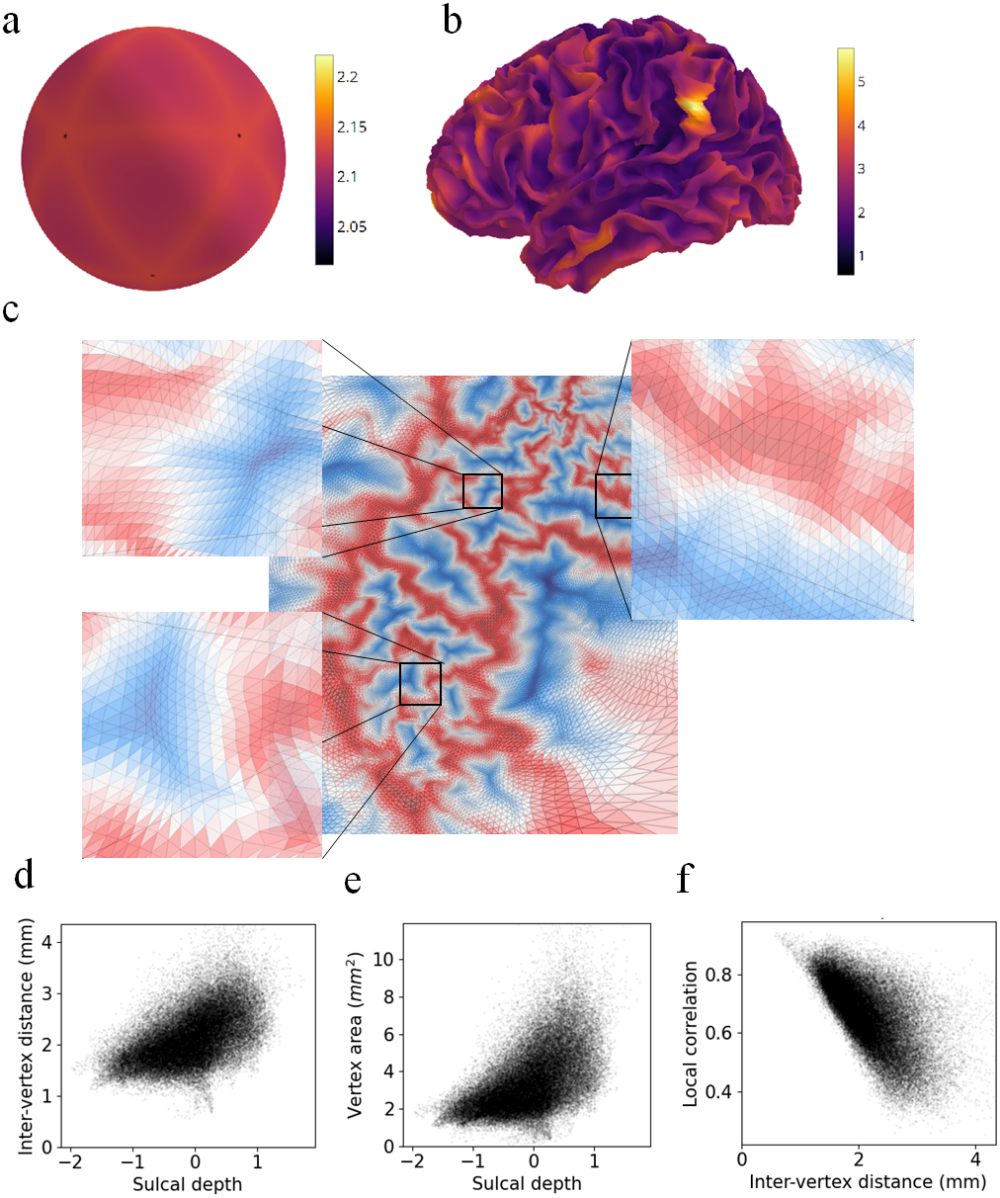
Inter-vertex distance and local correlations: (a) Inter-vertex distances on the inflated sphere. (b) Inter-vertex distances on the mid-thickness surface. (c) Visualisation of cortical surface mapped onto a 2D plane with a geometry-preserving transformation. Colours indicate gyri (red) and sulci (blue). Triangles represent mesh faces. (d) Inter-vertex distance on the mid-thickness surface plotted against sulcal depth. Higher sulcal depth values indicate gyri. (e) Vertex area plotted against sulcal depth. (f) Local fMRI correlation plotted against inter-vertex distance.

The primary reason for this bias in fMRI local correlations is that surface vertices are spaced further apart in gyri compared to sulci. Uneven spacing of vertices arises because evenly spaced vertices on the inflated sphere are mapped to unevenly spaced vertices on folded surfaces (seeSupplementary Methods 3for details). In a single participant, inter-vertex distances in the mid-thickness surface positively correlated with sulcal depth (r = 0.526, p < 0.001) (Fig. 3b-d). Results were similar when using nearest-neighbour distances instead of averaging across each vertex’s neighbours (r = 0.519, p < 0.001). Positive association between inter-vertex distances and sulcal depth was seen in all participants (mean r = 0.523; one-sample t-test, t(19) = 96.923, p < 0.001). Inter-vertex distances were directly visualised by mapping the cortical surface to a 2D plane with a geometry-preserving transformation (Supplementary Methods 2). All surface-to-plane projections unavoidably distort distances, but the transformation used minimised such distortions as far as possible. Distances were greater in gyri than in sulci, varying from about 1 mm at sulci to 3 mm at gyral crests (Fig. 3c). Vertex areas, being proportional to the square of inter-vertex distances, varied by an even greater factor (Fig. 3e). Resting-state fMRI correlation between a vertex and its immediate neighbours was negatively associated with inter-vertex distance (r = -0.570, p < 0.001) (Fig. 3f). The effect was present in every participant (mean r = -0.528; one-sample t-test t(19) = -41.183, p < 0.001). This appears self-evident, but many common analyses can inadvertently incorporate local correlations unadjusted for inter-vertex distances (seeSection 3.3).

There are two mechanisms by which unevenly spaced surface vertices lead to biased fMRI local correlations: (i) volume-to-surface mapping and (ii) surface smoothing.

#### 3.2.1 Volume-to-surface mapping

**Fig. 4.**
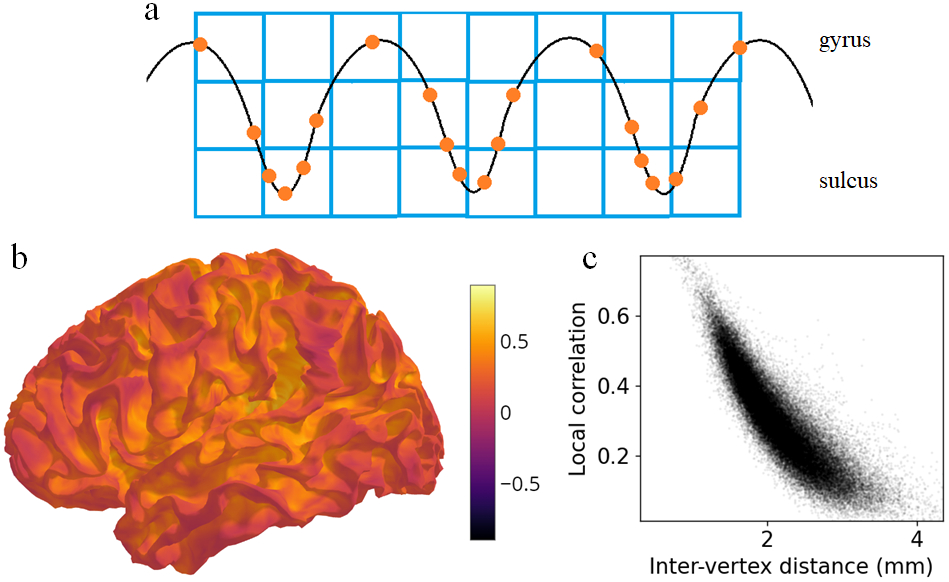
Volume-to-surface mapping induces artefactual local correlations. (a) Schematic showing that a single voxel’s (blue square) data are upsampled to multiple vertices (orange dot) due to small inter-vertex distances, especially in sulci. (b) Uncorrelated fMRI noise time series in volume MNI space were projected to the mid-thickness surface. This plot shows local correlations between adjacent vertices in the surface space. (c) Correlation between adjacent mesh vertices against inter-vertex distance.

When fMRI data is mapped from MNI volume space (2 mm voxels) to a high-resolution mesh such as the fsLR 32k surface, it is upsampled in some areas. In regions with low inter-vertex distances, such as sulci, one voxel is upsampled to more than one vertex (Fig. 4a). Thus, adjacent vertices will have the same or similar time series (Ciantar et al., 2022). This does not occur in regions with high inter-vertex distances such as gyri. To simulate this, we generated uncorrelated fMRI noise time series in MNI volume space, independently for each voxel. These time series were projected to a single participant’s mid-thickness surface using trilinear interpolation. The resulting surface time series had greater local correlation within sulci (Fig. 4b). Vertices with higher inter-vertex distance had lower local correlations (r = -0.856, p < 0.001) (Fig. 4c). Inter-vertex distance was associated with lower local correlations irrespective of the projection method (nearest neighbour, r = -0.622, p < 0.001; ribbon, r = -0.861, p < 0.001). The effect was present in every participant (mean r = -0.859; one-sample t-test, t(19) = -569.971, p < 0.001). Volume-to-volume mappings, for instance from native space to MNI space, could also potentially introduce spatially heterogenous upsampling and biased correlations. This can be explored in future work.

#### 3.2.2 Surface smoothing

**Fig. 5.**
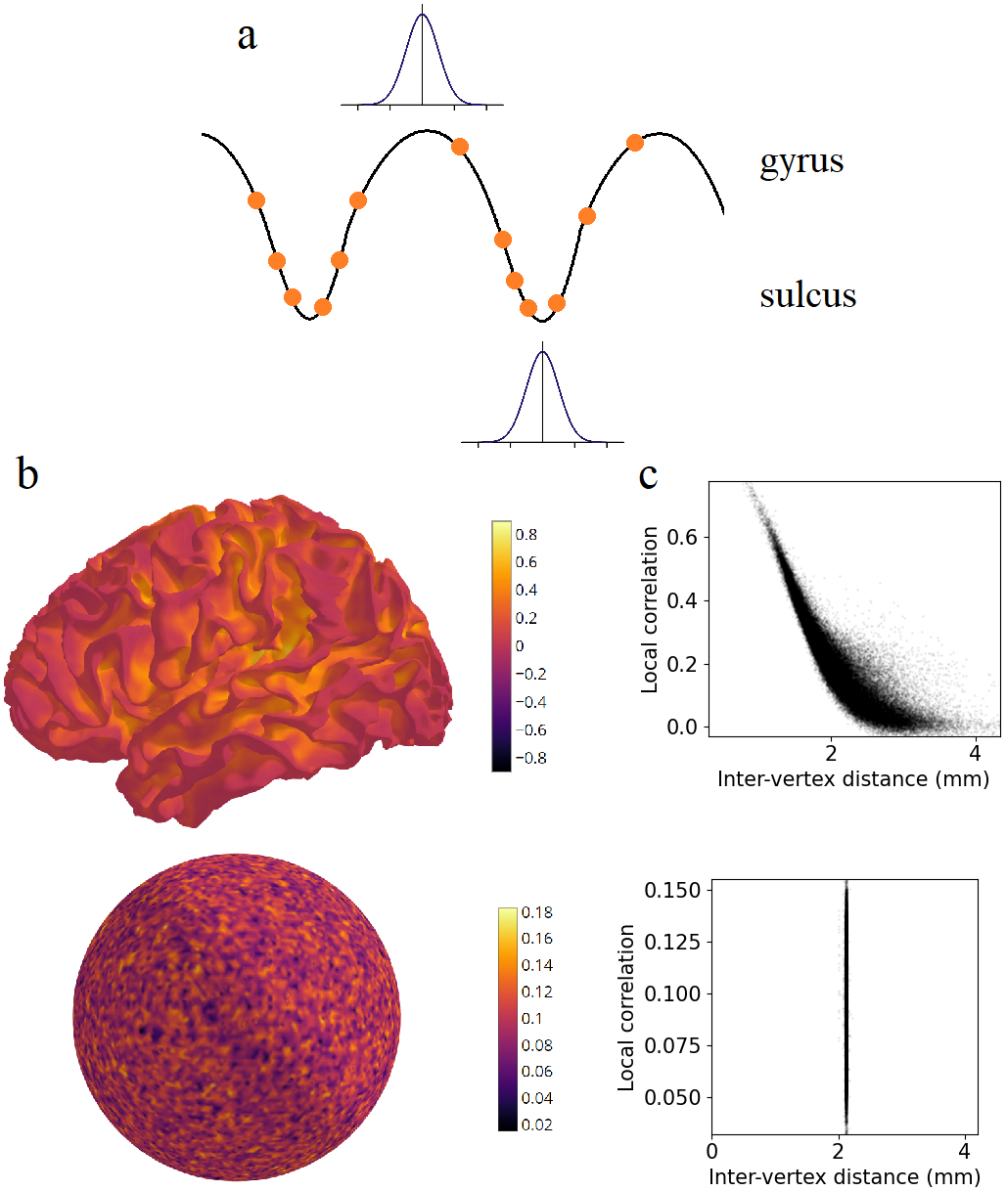
Surface smoothing induces artefactual local correlations. (a) Schematic showing that a Gaussian kernel of fixed width smooths over multiple cortical vertices to a greater extent in sulcal regions due to smaller inter-vertex distances. (b) Uncorrelated noise time series generated on the mid-thickness surface (top) or spherical surface (bottom) were smoothed with a 2 mm FWHM Gaussian kernel. This plot shows local correlations between adjacent vertices in the surface space. (c) Correlation between adjacent vertices against inter-vertex distance on the mid-thickness surface (top) or spherical surface (bottom).

Surface smoothing independently induces artefactual local correlations. Since vertices are closer to one another in sulci than in gyri, smoothing has greater effect on local correlations in sulci (Fig. 5a). To illustrate this, we generated uncorrelated noise time series, this time in fsLR 32k surface space, independently for each vertex. These time series were smoothed on the surface with a 2 mm FWHM Gaussian kernel, which is commonly used to smooth surface fMRI data (Glasser et al., 2013). This resulted in greater local correlation within sulci than in gyri (Fig. 5b), and a negative association between inter-vertex distance and local correlations (r = -0.812, p < 0.001) (Fig. 5c). There was no such bias when smoothing was applied to white noise generated on the spherical surface, since vertices are approximately evenly spaced on the sphere (r = 0.007, p = 0.208).

The artefactual correlations induced by volume-to-surface mapping can be avoided by using a lower resolution surface mesh (Ciantar et al., 2022). However, surface smoothing induces spurious local spatial structure even when a lower resolution mesh is used. In the group-averaged standard fsaverage5 pial surface, surface smoothing of noise data resulted in biased local correlations associated with inter-vertex distances (r = -0.790, p < 0.001) (Supplementary Fig. 2).

#### 3.2.3 Gyral bias in surface data does not originate from biases in volume data

It is plausible that differences in local correlations between gyri and sulci arise not from surface processing, but from earlier preprocessing steps, or from ground truth effects such as blood flow differences. We therefore tested for biases in empirical HCP resting-state fMRI volumetric data, where voxels are spaced uniformly in MNI space. For each voxel, mean correlation with its six neighbouring voxels (anterior, posterior, superior, inferior, left, right) were calculated. Voxels outside the grey matter were excluded. The map of local correlation values was calculated in volume space, then projected to the participant’s mid-thickness surface. Local correlations in volumetric space were not significantly associated with sulcal depth, irrespective of the projection method (trilinear interpolation r = 0.043, p = 0.297, nearest neighbour r = 0.034, p = 0.376, ribbon r = 0.030, p = 0.475) (Supplementary Fig. 3). This result suggests that the gyral bias in surface data arises from surface processing rather than being a feature of the BOLD response.

We next queried whether gyral bias on surface-mapped fMRI would persist if more distant vertices were considered, and if the data were adjusted for inter-vertex distances. To this end, empirical resting-state fMRI data were used to estimate, for each vertex, spatial autocorrelation as a function of distance. Spatial autocorrelation was not greater in sulci compared to gyri when adjusted for distance, rather than calculated amongst local vertices (Supplementary Results 1,Supplementary Fig. 4).

### 3.3 Consequences of gyral bias in functional fMRI

In the previous section, we demonstrated how uneven vertex spacing in the discretised cortical surface leads to artefactual correlations. In this section, we provide several examples of how fMRI gyral bias can contaminate surface-based neuroimaging analyses.

#### 3.3.1 Surface parcellations based on functional MRI

Functional parcellation schemes aim to divide the brain into contiguous parcels with relatively homogenous fMRI signals within each parcel. We hypothesised that the borders of a surface functional parcellation would be biased to prefer gyri over sulci. Parcel boundaries prefer areas where fMRI signal changes rapidly (gyri), while parcel centres tend to be located at vertices that are similar to many surrounding vertices (sulci).

To illustrate this, we constructed a null dataset for a single participant by generating random noise time series for each vertex on the participant’s fsLR 32k surface by sampling from a standard normal distribution. The time series had 500 time points. The data were smoothed by a 2 mm FWHM Gaussian kernel. The preceding analysis inSection 3.2.2demonstrated how this yields high local correlations in sulci. Thus, the correlations in the data implicitly contained participant-specific cortical folding information, rather than true functional information.

**Fig. 6.**
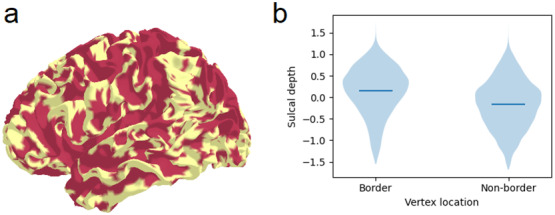
Boundaries of a functional parcellation are closer to gyri than to sulci. (a) Border vertices (pink) and non-border vertices (yellow) in one participant’s parcellation. (b) Sulcal depth values in border and non-border vertices. Positive sulcal depth values indicate gyri. Horizontal lines indicate mean.

We used a single participant’s null data constructed in this manner. Ward’s hierarchical agglomerative clustering algorithm (Johnson, 1967) was used to find 50 clusters in the left hemisphere (Thirion et al., 2014). After vertices were assigned to parcels, we identified border vertices as those which had a neighbour in a different parcel. Border vertices have a more positive mean sulcal depth value (0.145) compared to non-border vertices (-0.159), indicating that they are closer to gyri on average. The difference in sulcal depth values between border and non-border vertices was confirmed with a two-sample unpaired t-test (Cohen’s d = 0.540, t(29694) = 41.636, p < 0.001 with spin test) (Fig. 6). We then repeated the analysis in surrogate data generated on the surface of 20 other participants. Every participant had a positive t-statistic, indicating that border vertices were closer to gyri. A second-level one-sample t-test was employed to test whether the 20 participant-specific t-statistics significantly deviated from zero. The effect was significant (Cohen’s d = 5.479, t(19) = 23.882, p < 0.001), indicating that border vertices systematically align with gyri across participants.

We hypothesised that subject-specific cortical folding can contribute to the “individuality” in individualised functional parcellations. This is not necessarily the case, for example if functional parcel borders follow smoother group-averaged patterns of cortical folding but not individuals’ unique gyrifications. Following our hypothesis, we predicted border vertices calculated from one participant would not align with the gyri of a different participant. We tested the correspondence between the parcel borders of the 20 participants and the sulcal depth maps of 20*different*participants. This yielded a new set of 20 t-statistics. The new “mismatched” set of t-statistics were smaller than the original “matched” set (paired t-test, Cohen’s d = 2.240, t(19) = 21.051, p < 0.001), suggesting that individualised functional parcellations are influenced by the individual cortical folding information embedded in the local correlations of fMRI data.

We next tested whether these results held in commonly used group functional parcellations derived from empirical data. Parcel borders were closer to gyri in the Schaefer 300-node parcellation. Border vertices had greater sulcal depth values than non-border vertices (Cohen’s d = 0.127, t(29694) = 9.801, p < 0.001 with spin test). A second-level one-sample t-test confirmed that the effect was significant across all 20 participants (Cohen’s d = 6.431, t(19) = 28.031, p < 0.001). We then tested several other empirical parcellations. Border vertices were closer to gyri than sulci in the Schaefer 100-node and Glasser multi-modal parcellations (Supplementary Table 1). Conversely, border vertices were closer to sulci than gyri in older parcellations with anatomically derived boundaries: Desikan-Killiany and Harvard-Oxford. No such bias was seen in a randomly generated parcellation (Supplementary Table 1).

We then tested whether cortical folding influences the individuality of parcel boundaries in empirical individualised parcellations. In a commonly used 300-node individualised parcellation (Kong et al., 2021), an individual’s parcel boundaries followed the same individual’s cortical gyri more closely than the cortical gyri of a different individual (paired t-test, Cohen’s d = 1.266, t(19) = 5.848, p < 0.001).

#### 3.3.2 Test-retest reliability and fingerprinting in functional connectivity

“Fingerprinting” in fMRI refers to the ability to identify a given individual’s follow-up fMRI scan from within a large set of other participants’ scans, if one is provided the same individual’s baseline scan. Fingerprinting relies on low within-subjects variability (or high test-retest reliability) and high between-subjects variability.

It is known that cortical folding patterns are highly reliable within subjects and differ sufficiently between participants to allow fingerprinting (Pizzagalli et al., 2020). We have shown how local correlations track an individual’s cortical gyrifications. Therefore, vertex-resolution functional connectivity could potentially distinguish individuals by virtue of having structural information embedded in fine-scale correlation patterns.

As inSection 3.3.1, we used null data generated on the surface meshes of 20 different participants, constructed by generating spatially independent noise time series on the surface and smoothing it with a 2 mm FWHM kernel. Two such “scans” were generated for each participant, corresponding to test and re-test scans. Using these data, we investigated the fingerprinting accuracy of parcel-level and vertex-level functional connectivity separately. Individual parcel time series were obtained from a weighted average across vertices, where each vertex’s contribution was multiplied by the estimated vertex area. The 300 x 300 parcel-level functional connectivity matrix was calculated by taking the Pearson correlation between parcels’ time series. For the vertex level, we only considered vertices within a single parcel. This was because computing the full 59,412 x 59,412 vertex-level connectome would be computationally demanding, and unnecessary for our demonstration. We, therefore, computed functional connectivity within a single parcel in the 300-node parcellation. The time series for these vertices was represented by an N x 500 matrix, where N was the number of vertices within that parcel. The N x N vertex-level functional connectivity matrix was calculated using the Pearson correlation between vertex’s time series. Functional connectivity matrices were vectorised.

**Fig. 7.**
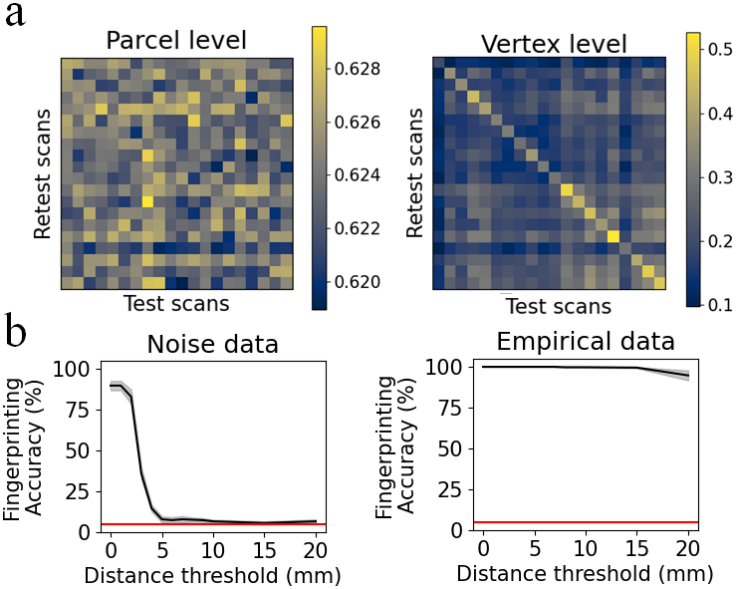
(a) Fingerprinting accuracy of parcel-level (left) and single-parcel vertex-level (right) functional connectivity in smoothed random noise data. Colours indicate the correlation coefficient between a participant’s test scan and a (potentially different) participant’s retest scan. Correlations were calculated between vectorised functional connectivity matrices. In each plot, each column corresponds to a different participant’s test scan. Each row corresponds to a different participant’s retest scan. (b) Fingerprinting accuracy of vertex-level functional connectivity in single parcels as a function of distance threshold, for smoothed random noise (left) or empirical resting-state fMRI (right). Functional connectivity data were omitted for vertices closer than the distance threshold. Bold line indicates mean fingerprinting accuracy across parcels. Shading indicates standard error of the mean. Horizontal line indicates chance level (5%).

Given a pair of participants A and B, we calculated the Pearson correlation between participant A’s test functional connectivity matrix, and participant B’s re-test functional connectivity matrix. This was repeated for all choices of participants A and B. Since test and re-test scans were generated as independent noise, the test-retest correlation should be similar irrespective of whether A = B or A ≠ B. This is the case for parcel-level functional connectivity. However, at the vertex level, each participant’s re-test scan was more strongly correlated with their own test scan than other participants’ test scans. We then sought to quantify fingerprinting accuracy. Each participant’s retest scan was matched with the test scan with which it was most highly correlated. Fingerprinting accuracy was quantified by the proportion of retest scans which were correctly matched. Parcel-level accuracy was 5%, equal to chance level. Vertex-level fingerprinting accuracy was calculated for each parcel separately, and averaged across all 300 parcels. Vertex level accuracy was greater than chance level (5%) in all parcels (mean 98%, minimum 75%) despite test and retest scans being uncorrelated noise (Fig. 7a).

We tested whether vertex-level fingerprinting accuracy depends on correlations between nearby vertices. Short-range connections were removed from the functional connectivity matrix. A threshold value for geodesic distance was selected. Elements of matrix F_V_^N^^x^^N^were removed if they corresponded to vertex pairs closer than the distance threshold. Fingerprinting accuracy dropped to chance level when the distance threshold was above 5 mm. In empirical resting-state data, fingerprinting accuracy remained high beyond the 5 mm distance threshold (Fig. 7b).

Considering this result, studies examining test-retest reliability and fingerprinting of fine-scale functional connectivity should be cautious of structural information embedded in fine-scale fMRI, especially when including correlations between nearby vertices. Heritability of fine-scale functional connectivity is another area of concern, as cortical folding patterns are heritable (Pizzagalli et al., 2020). Notably, fingerprinting of parcel-level functional connectivity derived from smoothed random noise is no higher than chance.

#### 3.3.3 Sulcal vertices contribute disproportionately to parcels

Parcel-level analyses rely on averaging the values of all vertices within a parcel. We have shown that inter-vertex distances in gyri are two to three times greater than in sulci. This means that sulcal vertices are over-represented in surface space. In some implementations, such as the Connectome Workbench, computing a parcel’s time series involves weighting each vertex’s contribution by its surface area (Marcus et al., 2011). However, studies often compute parcel-mean values by averaging across vertices without correcting for surface area (Byrge & Kennedy, 2018;Kashyap et al., 2023;Qiu et al., 2022;Schaefer et al., 2018).These parcel mean values will under-represent gyral data. This issue is relevant to analyses that employ vertex-level data, including fMRI, vertex-wise genetic maps, or spatial maps of myelin thickness. As an illustration, we generated a single participant’s null data as inSection 3.3.1by surface smoothing a spatially independent white noise time series. Parcel mean time series were calculated for each parcel in the Schaefer 300-node parcellation. For each vertex, we calculated the Pearson correlation between its time series and the time series of its encompassing parcel. This reflects the contribution of each vertex to its supervening parcel. We tested whether a vertex’s sulcal depth was associated with its correlation with the parcel mean. Gyral vertices in this test data had significantly lower correlations with parcel means compared to sulcal vertices (r = -0.215, p < 0.001 with spin test), indicating that they are under-represented at the parcel level (Supplementary Fig. 5). The effect was present in every participant (mean r = -0.224, one-sample t-test, t(19) = -54.558, p < 0.001). Parcel mean time series were then re-calculated using a weighted average, where each vertex’s value was multiplied by the estimated vertex area. This procedure reduced the magnitude of the bias but did not abolish it, (r = 0.101, p < 0.001 with spin test). A similar result was seen in all participants (mean r = 0.085, t(19) = 22.529, p < 0.001).

Biased parcel means occurred because uncorrelated noise time series in sulcal vertices became correlated after surface smoothing. Gyral vertices remained relatively uncorrelated because they are distant relative to the 2 mm FWHM smoothing kernel. When parcel means are calculated, the time series of uncorrelated gyral vertices tend to cancel out. Conversely, the time series of correlated sulcal vertices contributed collectively to the parcel average. This example demonstrates that sulcal vertices can contribute disproportionately to surface-based parcel-level analyses.

### 3.4 Onavg template

The new standard onavg template has substantially lower variability in inter-vertex spacing, and should have lower gyral bias. However, projecting the standard onavg template to participant-specific anatomically idiosyncratic surfaces could distort vertex spacings, potentially increasing the variance in inter-vertex spacing. Therefore, we tested for gyral bias in the participant-specific onavg surface. Participant-specific onavg-ico32 surfaces were generated for all 20 participants (Supplementary Methods 4). Following the original paper, the onavg-ico32 surface was compared to the fsaverage5 surface as both had 10,242 vertices per hemisphere. The standard deviation of inter-vertex distances was 20.7% lower in onavg (mean SD = 0.713) than in fsaverage5 (mean SD = 0.899) (paired t-test, t(19) = -42.651, p < 0.001), a more modest reduction than previously reported in standard meshes (Feilong et al., 2023). We tested for association between inter-vertex distance and sulcal depth (as inSection 3.2). The association was reduced in onavg (mean r = 0.087) compared to fsaverage5 (mean r = 0.326) (paired t-test, t(19) = -44.825, p < 0.001). We then evaluated the effect of surface smoothing on local correlations (Section 3.2.2) using a 4 mm FWHM kernel because of greater inter-vertex distances in these surfaces compared to fsLR 32k. The inverse association between sulcal depth and local correlations in the fsaverage5 surface (mean r = -0.328) was absent in onavg-ico32 (mean r = 0.012) (paired t-test, t(19) = -57.287, p < 0.001). Next, we tested for gyral bias in functional parcel boundaries using noise data (Section 3.3.1). Across 20 participants, the bias (quantified by t-statistic) was significantly reduced in onavg-ico32 (mean t-statistic -0.761) compared to fsaverage5 (mean t-statistic 10.887) (Cohen’s d = 4.282, paired t-test, t(19) = -15.113, p < 0.001). Testing whether a vertex’s sulcal depth was associated with its correlation with the parcel mean (Section 3.3.3), we found that biased parcel means were reduced with onavg-ico32 (mean r = -0.077) compared to fsaverage5 (mean r = -0.192) (paired t-test, t(19) = -29.746, p < 0.001).

However, vertex-level fingerprinting accuracy of noise data (Section 3.3.2) was greater for onavg-ico32 (mean accuracy across parcels 48%) than fsaverage5 (mean 42%), (paired t-test, t(299) = 3.653, p < 0.001). We sought to understand this surprising result. Inflated fingerprinting accuracy in noise-derived functional connectivity matrices arises from variability in the spatial map of inter-vertex distances. More specifically, it originates from the component of variability that is unique and not shared across individuals. In fsaverage5, variability in inter-vertex distances arises from the gyral bias. Larger cortical folds such as the central sulcus are common across individuals, so a large part of the variance is shared across individuals, and hence does not contribute to fingerprinting accuracy. We hypothesised that vertex-level fingerprinting is greater in onavg because a greater fraction of the variability is participant-specific rather than being associated with large cortical folds. We tested this by calculating the distance between each vertex and its nearest neighbour. This yielded a spatial map of nearest-neighbour distances for each participant. The correlation between different participants’ spatial maps was lower in onavg (mean r = 0.145) than in fsaverage5 (mean r = 0.239) (paired t-test, t(299) = -12.597, p < 0.001), indicating that the spatial map is more individual-specific in onavg.

## 4 Discussion

We studied the influence of heterogenous vertex spacing in commonly used surface meshes, which possess greater inter-vertex distances in gyri compared to sulci. This gyral bias in inter-vertex distances yields a gyral bias in nearest-neighbour correlations. In particular, uneven vertex spacing and local correlations track not only group-averaged cortical folding, but also individuals’ gyrifications.

This manuscript departs from previous work in several aspects. Previous examinations of these geometric biases primarily used standard mesh surfaces, where local correlations appeared mostly to track the major cortical folds such as the central sulcus and postcentral sulcus (Ciantar et al., 2022). We used a larger sample size (n = 20) than this previous work. Notably, the stronger association between a participant’s sulcal depth and their own local correlation map compared to the next individual’s local correlation map was present in every participant (Fig. 2e). However, reproducibility and robustness could be further improved with a larger sample size. We also confirmed that biased local correlations do not exist in original volumetric data, and demonstrated that local correlations can be biased not only by volume-to-surface mapping as seen in previous work (Ciantar et al., 2022), but also by surface smoothing. Finally, we showed potential consequences of biased correlations. Individually, specific anatomical information can leak into functional data, resulting in biases in individualised parcellations and spurious fingerprinting results. In smoothed synthesised noise data, the spatial pattern of fMRI correlations derives from the location of cortical gyri and sulci. Consequently, fMRI correlations have high test-retest reliability, contributing to high vertex-level fingerprinting. Future work could also assess fingerprinting accuracy using serial structural scans in the same individual (Gordon et al., 2017;O’Connor et al., 2017), to evaluate the test-retest reliability of cortical folding patterns. Parcel-level fingerprinting of random data was not above chance level, indicating that parcel-level fingerprinting studies (Finn et al., 2015) are less likely to be affected by this bias. We urge caution about vertex-level analyses, especially as tools to perform vertex-resolution MRI analysis are increasingly available (Loewe et al., 2016;Mansour L et al., 2022).

In many commonly used surface spaces, vertices are positioned more densely in sulci, with sulci being oversampled by a factor of 3 or more relative to gyri. This stems from preprocessing steps that have been standardised across the field (Supplementary Methods 3). Consequently, surface-based functional parcellations can be susceptible to fMRI gyral bias. Most functional parcellations directly cluster vertex time series, use fMRI connectivity gradients to find parcel boundaries, or combine both approaches. Direct clustering leads to parcel centroids that are biased towards sulci, because sulcal data are over-represented. fMRI connectivity gradients, when uncorrected for inter-vertex distances, rapidly change in gyri due to low local correlations, leading to parcel boundaries biased towards gyral loci. In vertex-level functional connectivity, we found that smoothed noise time series are spuriously identifiable if correlations between nearby vertices (within 5 mm) are included. We explain the 5 mm threshold by noting that it is approximately equal to the largest inter-vertex distances on the fsLR 32k cortical mesh (Fig. 3). Some gyral vertices are 5 mm from their nearest neighbours, while sulcal vertices have much closer nearest neighbours. Omitting short-range connections removes this distinguishing feature of sulcal vertices and reduces the influence of cortical folding patterns on fMRI correlations. In general, we advise researchers against calculating fMRI correlations between vertices that are within a critical distance threshold, that is, the largest inter-vertex geodesic distance, which is about 5 mm for the fsLR 32k mesh.

There are several other scenarios where analyses can be confounded by these biases. These fall into two categories—situations arising from oversampling of sulci and those arising from embedding structural information in fMRI correlations.

There are several other possible consequences of oversampling sulci. First, relative under-sampling of gyri could impact predictive accuracy in multi-voxel pattern analysis or brain-behaviour prediction. Second, it can lead to problems with hyperalignment (Haxby et al., 2011,^2020^) (also known as functional alignment), especially when applied on surface meshes (Busch et al., 2021;Feilong et al., 2021;Guntupalli et al., 2018). This method estimates a transformation matrix that maps vertices in one participant to vertices in another. The optimal matrix transforms the first participant’s functional response to predict the second participant’s response. There can be constraints on the matrix’s form. For instance, a permutation matrix maps single vertices to single vertices. In optimal transport, each vertex contributes equal “mass” that is divided among target vertices in the output space (Bazeille et al., 2019). These approaches assume the equivalence of gyral and sulcal vertices. The assumption is not substantiated in surface meshes, where a gyral vertex typically occupies a surface area that is the equivalent of multiple sulcal vertices.

Confounding of results can also arise from structural information embedded in the local correlations of fMRI. While we have shown artefactual gyral biases in surface fMRI data, similar biases may be present in other modalities, influencing any associations between fMRI and data from other modalities. Some examples of biases in other modalities are as follows. First, cortical thickness has been found to be greater in gyri (Fischl & Dale, 2000).Second, tractography algorithms in diffusion MRI terminate streamlines preferentially at gyri (Schilling et al., 2017). Third, EEG and MEG are more sensitive to gyral source and sulcal walls respectively due to different orientations of the dipole fields (Srinivasan, 2006). Hence, testing for associations between these different modalities must be approached with caution. Regional homogeneity (Jiang & Zuo, 2016), which is a measure of fMRI correlation between neighbouring vertices, would also be artefactually elevated in sulci when calculated on surface fMRI data. Surface-based regional homogeneity changes have been previously found in schizophrenia (Cao et al., 2021;Zhang et al., 2017), autism spectrum disorder (Jiang et al., 2015), eating disorders (Wang et al., 2023), cognitive impairment (Zhang et al., 2017), and in association with personality traits (Li et al., 2017). The distribution of fMRI correlations across the cortex could be influenced by several other factors, including regional differences in fMRI signal-to-noise ratio (Jezzard & Clare, 1999), head motion, and the distribution of vasculature (Huck et al., 2023).

Finally, when predictive models are trained on vertex-resolution fMRI to predict behaviour or cognitive phenotypes, implicit structural information may be contributing to the prediction. For instance, a neural network may learn to use the correlation between adjacent vertices (which tracks cortical folding) as a feature for prediction.

There are several approaches to excluding fMRI gyral biases. First, using a surface mesh with resolution less than that of the voxel data avoids the problem of upsampling during volume-to-surface mapping (Ciantar et al., 2022). For example, projecting from 2 mm MNI voxel space to the coarse fsaverage5 surface (with 10 k vertices) avoids projecting one voxel onto multiple vertices. However, even at these lower resolutions, surface smoothing will induce artefactual local correlations. Another possibility is to conduct analyses such as parcellation in volume space before projecting results to the surface (Ciantar et al., 2022). This eschews the benefits of surface representations. For instance, a volume-based parcellation could misconstrue voxels on opposite sulcal banks as adjacent. This issue can be addressed with a hybrid approach, conducting analyses in volume space, but with voxel neighbours constrained by the cortical surface (Ciantar et al., 2022). A recent promising approach has been the introduction of surface templates with more homogenous inter-vertex distances. Uniform meshes could potentially reduce the impact of “problem areas” with outlier inter-vertex distances (Ciantar et al., 2022). The standard onavg template, developed through an iterative procedure of adjusting vertex locations, substantially reduces variability in inter-vertex spacing compared to standard meshes (Feilong et al., 2023). On participant-specific onavg surfaces, the reduction in variability in inter-vertex distances was more modest (20.7%), likely due to inter-individual differences in cortical folding. On participant-specific surfaces, the onavg template reduced gyral biases in inter-vertex distances, local correlations, functional parcellations, and parcel-averages. Artefactual inflation in vertex-level fingerprinting was not improved by the onavg template, likely due to the relatively high residual individual-specific variability in inter-vertex distances. A “perfect” group-level template is impossible because vertex locations with uniform inter-vertex distances in one participant would not have the same uniform spacings in another. For within-participant analyses like multi-voxel pattern analysis, generating participant-specific meshes using the same iterative method (Feilong et al., 2023) may be the best option, which could be tested in future work. Even with uniform templates, voxel subsampling should be avoided by using a template with lower resolution than the underlying volume data.

We acknowledge that excluding the fMRI gyral bias is an ongoing challenge. Hence, researchers should use quantitative measures that are robust against uneven vertex spacing. For instance, spatial autocorrelation as a function of geodesic inter-vertex distance is robust, while nearest-neighbour correlation is not robust to this bias. We recommend that parcel means should use vertex area weighted averages to reduce the practical significance of the bias. Even with these modifications, the influence of fMRI gyral biases may remain obscure in complex multi-step pipelines. In this situation, researchers can still test whether fMRI gyral bias can explain study results. By running a pipeline on gyrally biased random noise data instead of empirical data, one can test whether the observed finding arises spuriously from randomly generated null models. Surface-smoothed Gaussian noise data as studied here can be used for this purpose. Finally, any study results should be visualised on participant-specific non-inflated surfaces, so that obvious covariation with sulcal depth can be identified.

In summary, cortical data benefit greatly from surface representations due to the cortex’s folded sheet-like topology. However, researchers should remember that cortical meshes use a somewhat arbitrary tessellation to discretise the brain surface. Care should be taken to ensure that the chosen analysis does not implicitly treat vertices as ground truth entities or features.

## Supplementary Material

Supplementary Material

## Data Availability

Code to perform analyses is available athttps://github.com/jaysonjeg/track_align. Code to map the onavg template to participant-specific surfaces is available athttps://github.com/nikitas-k/onavg-ind/, function nativeMNI_to_onavg in script register.py. Data were from the Human Connectome Project and can be accessed athttps://db.humanconnectome.org/
