# Supplementary material for: Spurious correlations in surface-based functional brain imaging
Source: Imaging Neurosci (Camb). 2025 Feb 18;3:imag_a_00478. doi: 10.1162/imag_a_00478 (PMC12319870; doi:10.1162/imag_a_00478)
Supplement: Supplementary Material [file imag_a_00478-supp.pdf]

Supplementary Materials

Supplementary Methods 1. HCP participant IDs

The first 20 participants with both 3T and 7T data were used. These participants had the following IDs: 100610, 102311, 102816, 104416, 105923, 108323, 109123, 111514, 114823, 115017, 115825, 116726, 118225, 125525, 126426, 128935, 130114, 130518, 131722, 134829.

Supplementary Methods 2. Geometry-preserving mapping from cortical surface to 2D plane

The cortical surface without the medial wall can be topologically simplified to a connected open surface. Such surfaces are equivalent to geometric forms like squares, rectangles, or disks. The transformation from a 3D cortical surface to a planer 2D surface was performed with quasi-conformal mapping (Meng et al., 2016). This mapping allows for detailed feature analysis while preserving local geometry by solving the Beltrami equation: $\frac{\partial f}{\partial \bar{z}} = \mu(z) * \frac{\partial f}{\partial z}$. Here, f represents the mapping function transforming coordinates from one complex domain (the cortical surface) to another (a 2D plane). The complex-valued function $\mu(z)$, known as the Beltrami coefficient, quantifies the local distortion inherent to the mapping. This preservation of local geometry is critical for maintaining the fidelity of anatomical features during the transformation. Specifically, the original triangulation of the cortical mesh was preserved during the mapping from 3D cortical surface onto a 2D plane. The resulting 2D cortical mesh, constructed with the mapped vertices and faces, preserves the underlying topology of the original 3D cortical surface, and consequently provides a visual representation of cortical topological features.

Supplementary Methods 3. FreeSurfer and fsLR spherical registration and downstream gyral bias

The FreeSurfer spherical registration method offers several advantages over volumetric registration, with a robust method for inter-subject registration of fMRI studies. After cortical reconstruction of pial and white surfaces, these are inflated using an energy-minimization algorithm (Fischl et al., 1999) that minimizes metric distortions of the surfaces. These participant-specific spheres are then registered to the FreeSurfer spherical template space, allowing alignment to a common space for intersubject comparison. However, the transformation of cortical surfaces to this common space induces a gyral bias. Vertices are nearly uniformly spaced on the FreeSurfer template sphere, but this corresponds to unevenly spaced vertices in the subsequent folded cortical surfaces. This arises from the method of "re-folding" the cortex from the sphere.

As an example, we highlight the steps in the HCP Minimal Preprocessing Pipeline that are involved in these transformations (Glasser et al., 2013). The pipeline distinguishes a *Native* coordinate space that is specific to the subject's cortical reconstructions; these reconstructions being derived from FreeSurfer prior to FreeSurfer spherical registration (i.e., using *recon-all* up to --*autorecon3*). These reconstructions have unevenly spaced vertices but express no discernible pattern to this spacing. In the unmodified *HCPpipelines* post-FreeSurfer registration step contained here: https://github.com/Washington-University/HCPpipelines/blob/master/PostFreeSurfer/scripts/FreeSurfer2CaretConvertAndRegisterNonlinear.sh, with default parameters, *Native* surfaces are saved to the filepath "{Subject}/T1w/Native". Registration of these surfaces to the *fsLR* template then proceeds to a (i) high-resolution *164k* space with 163,842 vertices per hemisphere, equivalent to

the *fsaverage* space (also with 163,842 vertices; however, these were reordered in *fsLR* to be roughly equivalent across hemispheres (Van Essen et al., 2012), and to a (ii) low-resolution *32k* space, with 32,492 vertices per hemisphere.

Both template spheres, *164k* and *32k*, have nearly uniform inter-vertex spacing. The (i) registration to the fsLR *164k* template utilises the FreeSurfer template-registered individual subject spheres (copied from the individual's FreeSurfer "{l/r}h.sphere.reg" to "{Subject}/MNINonLinear/{Subject}.{Hemisphere}.sphere.164k_fs_LR.surf.gii"). As these spheres are registered to the FreeSurfer template space, the gyral bias makes its way to the subject's "MNINonLinear" surfaces. These surfaces are contained in the filepath "{Subject}/MNINonLinear/", "{Subject}/MNINonLinear/Native".

Registration in (ii) proceeds by *Native* registration to the *32k* downsampled template sphere. The definition of vertex points in the *32k* template sphere was obtained similarly to the *fsaverage* template spheres. The template sphere was parameterized as a geodesic polyhedron (in this case, a subdivided icosahedron with a spacing of 2.02-2.18 mm; see Figure 3a). In the HCP Pipelines (with default parameters) this template sphere is contained in https://github.com/Washington-University/HCPpipelines/blob/master/global/templates/standard_mesh_atlases/L.sphere.32k_fs_LR.surf.gii. This file is copied to "{Subject}/MNINonLinear/fsaverage_LR32k/{Subject}.{Hemisphere}.sphere.32k_fs_LR.surf.gii" during the *PostFreeSurfer* pipeline. While the points are evenly spaced on the spherical surface, the sampling of points in the resultant *32k* folded cortical surfaces is far denser in sulci than gyri.

Supplementary Methods 4. Generating participant-specific onavg-ico32 and fsaverage5 surfaces

Each participant's unique cortical surface can be tessellated into vertices and triangles in different ways. Possible tessellations vary in density and resolution, and in the uniformity of inter-vertex spacing. A participant's surface can be inflated to a sphere to represent the tessellation on a common spherical surface. In reverse, a "template" tessellation on the sphere can be deflated onto participant-specific cortical surfaces. Resampling vertices between different template tessellations, for instance from the fsaverage surface to the onavg surfaces, is straightforward because both templates are represented on a common spherical surface.

To generate participant-specific onavg-ico32 surfaces, we used the function -surface-resample in Connectome Workbench, where the participant's MNINonLinear native surface was directly resampled onto the onavg surface (or the fsaverage5 surface for comparison). The other inputs were the original FreeSurfer sphere "lh.sphere.reg" and the target onavg sphere "tpl-onavg_hemi-L_den-10k_sphere.surf.gii". The latter is in register with the FreeSurfer sphere, so resampling is straightforward.

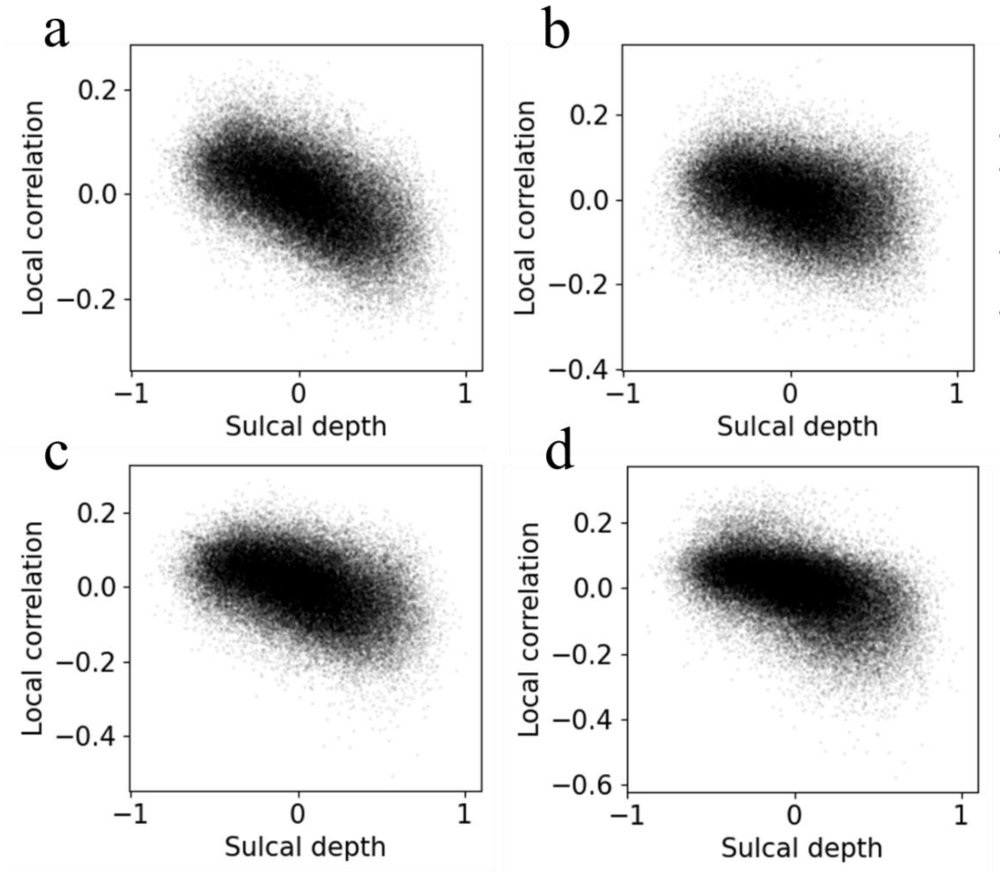

Supplementary Figure 1. fMRI local correlations plotted against sulcal depth. Higher sulcal depth values indicate gyri. Both variables were normalized by parcel-specific mean values. fMRI local correlations tracked individuals' unique cortical folding, irrespective of changes to the fMRI data type. a) Using 4 runs of resting-state fMRI (1 hour) instead of 1 run (15 minutes) (r=-0.544, p<0.001). b) Using resting-state data aligned with MSMAll instead of MSMSulc (r=-0.302, p<0.001). c) Using 7T movie viewing fMRI (1 run) (r=-0.419, p<0.001). d) Using the correlation between each vertex and its single nearest neighbour instead of averaging across each vertex's neighbours (r=-0.460, p<0.001).

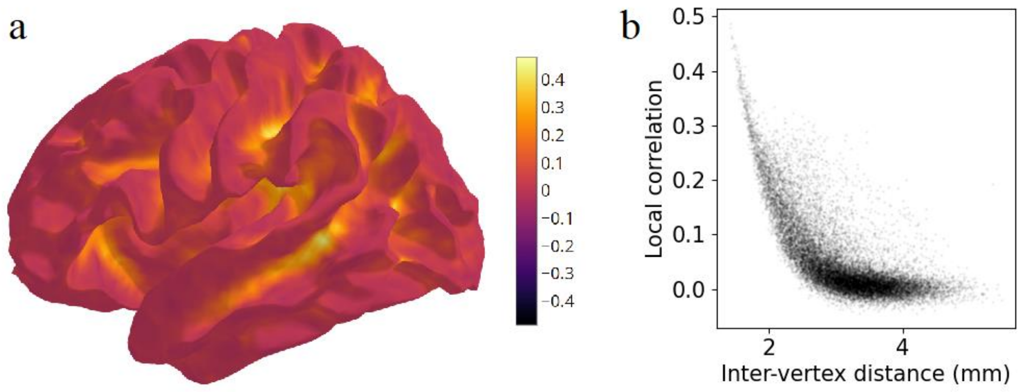

Supplementary Figure 2. Surface smoothing (2 mm FWHM Gaussian kernel) induces biased fMRI correlations in the fsaverage5 pial surface. Uncorrelated noise was generated at each surface vertex. a) Local correlation at each vertex. b) Local correlation plotted against inter-vertex distance (r=-0.790, p<0.001).

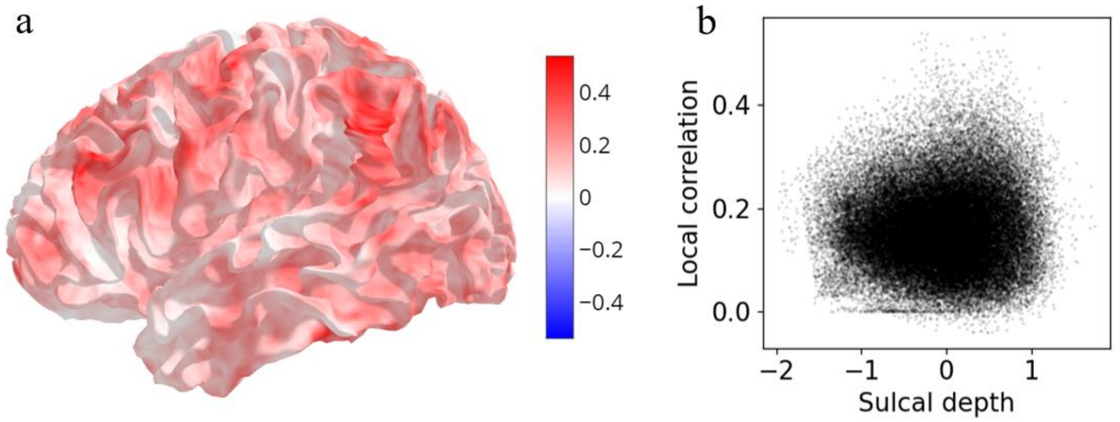

Supplementary Figure 3. Local correlations in resting-state fMRI in volume space, which were then projected to the surface with trilinear interpolation . a) Local correlations. b) Local correlation plotted against sulcal depth (r=0.043, p=0.297). Higher sulcal depth values indicate gyri.

<u>Supplementary Results 1. Spatial autocorrelation as a function of distance</u>

We used empirical HCP resting-state fMRI data in the fsLR 32k surface space. The following pipeline was followed to estimate a different spatial autocorrelation function for each vertex. The original vertex was termed the “source vertex”. We considered an expanded definition of vertex neighbourhood, and all other vertices within a 10 mm geodesic distance were termed “neighbours”. We calculated the fMRI correlation between the source vertex time series and its neighbours’ time series, and the geodesic correlation between them on the mid-thickness mesh. These data characterized the source vertex’s spatial autocorrelation function. An exponential curve $y = Ae^{-kx} + c$ was fitted to predict correlation from distance. Parameter k, corresponding to the decay rate, indicates how rapidly spatial autocorrelation drops with distance from the source vertex. We calculated the decay rate for each vertex on the fsLR 32k surface. The decay rate is reduced in gyri compared to sulci (r=-0.190, p<0.001), indicating that the spatial extent of spatial autocorrelation is larger in gyri (Supplementary Figure 4).

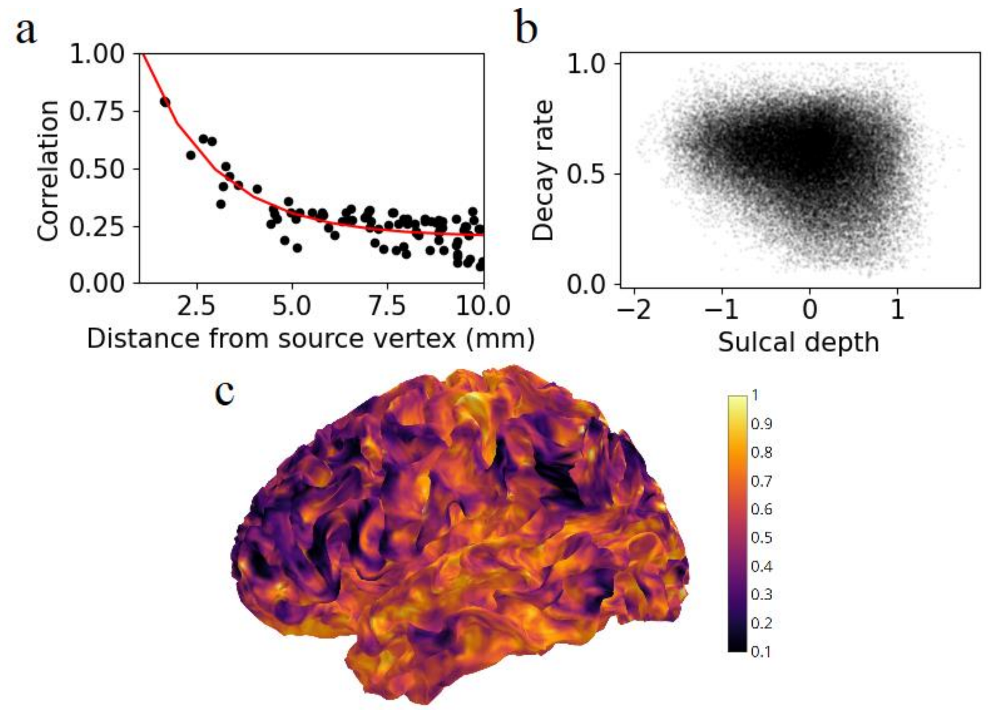

Supplementary Figure 4. Spatial autocorrelation as a function of distance on the mid-thickness surface. a) Example of the spatial autocorrelation function of a single vertex. The red line indicates the fitted exponential function b) The exponential function decay rate is plotted against sulcal depth. Each data point indicates a single vertex. Positive sulcal depth values indicate gyri. c) Spatial map of the decay rate.

**Parcellation**	**t-statistic (df=19)**	**p-value**
Brodmann	-16.859	<0.001
Desikan-Killiany	-50.081	<0.001
Harvard-Oxford	-37.382	<0.001
Glasser multi-modal	6.642	<0.001
Schaefer 300-node	28.031	<0.001
Schaefer 100-node	34.973	<0.001
k-means 300-node	-0.502	0.621

Supplementary Table 1. Gyral bias in empirical functional parcellations. Volumetric parcellations such as the Harvard-Oxford atlas were projected to the fsLR 32k surface before analysis. The k-means parcellation was derived from k-means clustering of vertices based on their (x,y,z) coordinates. For each parcellation and each participant (n=20), a two-sample t-test compared the mean sulcal depth of border and non-border vertices. For each parcellation, a second-level one-sample t-test (across participants) assessed whether the first-level t-statistics deviated significantly from zero. A positive t-statistic indicates that border vertices were closer to gyri than to sulci.

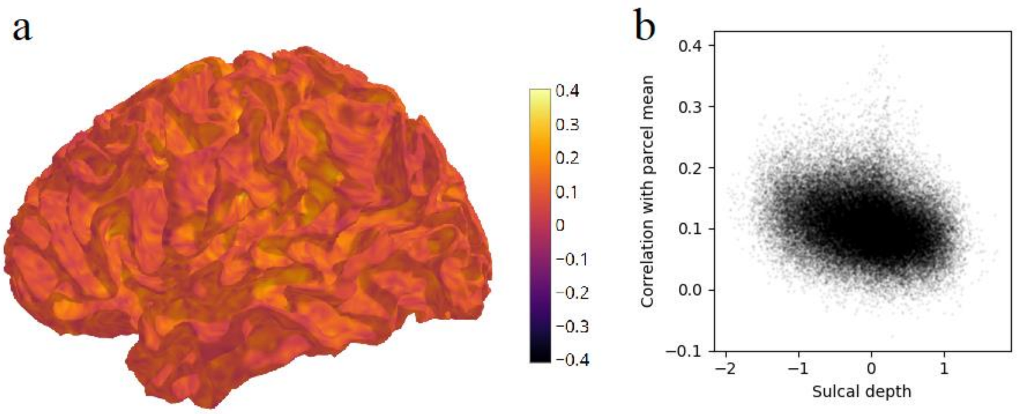

Supplementary Figure 5. Sulcal vertices contribute disproportionately to parcel means. Noise time series were smoothed with a 2 mm FWHM Gaussian kernel. a) Correlation between each vertex's time series and its parcel mean time series. b) Plot of the correlations in (a) against sulcal depth. Positive values indicate gyri. $r=-0.215$, $p<0.001$

References

Fischl, B., Sereno, M. I., & Dale, A. M. (1999). Cortical Surface-Based Analysis: II: Inflation, Flattening, and a Surface-Based Coordinate System. *NeuroImage*, *9*(2), 195–207. https://doi.org/10.1006/nimg.1998.0396

Glasser, M. F., Sotiropoulos, S. N., Wilson, J. A., Coalson, T. S., Fischl, B., Andersson, J. L., Xu, J., Jbabdi, S., Webster, M., Polimeni, J. R., Van Essen, D. C., Jenkinson, M., & WU-Minn HCP Consortium. (2013). The minimal preprocessing pipelines for the Human Connectome Project. *NeuroImage*, *80*, 105–124. https://doi.org/10.1016/j.neuroimage.2013.04.127

Meng, T. W., Choi, G. P.-T., & Lui, L. M. (2016). TEMPO: Feature-Endowed Teichmüller Extremal Mappings of Point Clouds. *SIAM Journal on Imaging Sciences*, *9*(4), 1922–1962. https://doi.org/10.1137/15M1049117

Van Essen, D. C., Glasser, M. F., Dierker, D. L., Harwell, J., & Coalson, T. (2012). Parcellations and hemispheric asymmetries of human cerebral cortex analyzed on surface-based atlases. *Cerebral Cortex (New York, N.Y.: 1991)*, *22*(10), 2241–2262. https://doi.org/10.1093/cercor/bhr291
